# Supplementary material for: Early Recovery of Left Ventricular Function After Revascularization in Acute Coronary Syndrome
Source: J Clin Med. 2019 Dec 20;9(1):24. doi: 10.3390/jcm9010024 (PMC7019788; doi:10.3390/jcm9010024)
Supplement: Supplementary file 1 [file jcm-09-00024-s001.pdf]

Supplement 1. Clinical and biochemical data.

Variable	Patients (n = 80)
Age (years)	5.7 ± 9.4
Gender (female, n %)	21 (23)
Single vessel disease (n, %)	53 (66)
Two vessel disease (n, %)	12 (15)
Multi vessel disease (n, %)	15 (19)
Smoking (n, %)	51 (63)
Diabetes (n, %)	32 (40.1)
Angina class I (n, %)	45 (56)
Angina class II (n, %)	23 (29)
Angina class III (n, %)	12 (15)
Dyslipidemia (n, %)	19 (23.8)
Positive family (n, %)	0 (0)
HTN (n, %)	49 (51)
SBP (mmHg)	130.6 ± 20
DBP (mmHg)	79 ± 10
HR (beats/min)	78 ± 12
ECG abnormality (n, %)	63 (78)
Abnormality in LAD territories (n, %)	58 (72.5)
Abnormality in LCx territories (n, %)	34 (42.5)
Abnormality in RCA territories (n, %)	0 (0)
Troponin ()	22.2 ± 22
CK-MB (UI/L)	104.2 ± 110
Creatinine ()	0.95 ± 0.19
Hemoglobin (g/dL)	13.5 ± 1.4

SBP: systolic blood pressure; DBP: diastolic blood pressure; HR: heart rate; DM: Diabetes mellitus; HTN: Hypertension; ECG: Electrocardiography; CK-MB: Creatine kinase-MB.

Supplement 2. Echocardiographic & Electrocardiographic date.

Variable	Patients (n = 80)
Echocardiographic dimension	
LV EDD (cm)	53 ± 5.1
LV ESD (cm)	3.2 ± 0.4
IVSd (cm)	1.7 ± 0.14
LVPWd (cm)	1.1 ± 0.1
LV EF (%)	57 ± 10
LA (mm)	35.7 ± 5.1
Aorta (mm)	27.6 ±5.5
E/A ratio normal (n, %)	31 (38.8)
E/A ratio reserved (n, %)	49 (61.3)
Electrocardiographic abnormality	63 (78%)
Abnormality on LAD derivation (n, %)	58 (72.5)
Abnormality on LCx derivation (n, %)	34 (42.5)
Abnormality on RCA derivation (n, %)	0 (0)

LV: left ventricle; EDD: end-diastolic dimension; ESD: end-systolic dimension; IVSd: inter-ventricular septum in diastole; PWd: parietal wall in diastole; EDV: end-diastolic volume; ESV: end systolic volume; LA: left atrial; MR: mitral regurgitation; AR: aortic regurgitation; PR: pulmonary regurgitation; LAD: left anterior descending artery; LCx: left circumflex artery; RCA: Right coronary artery.

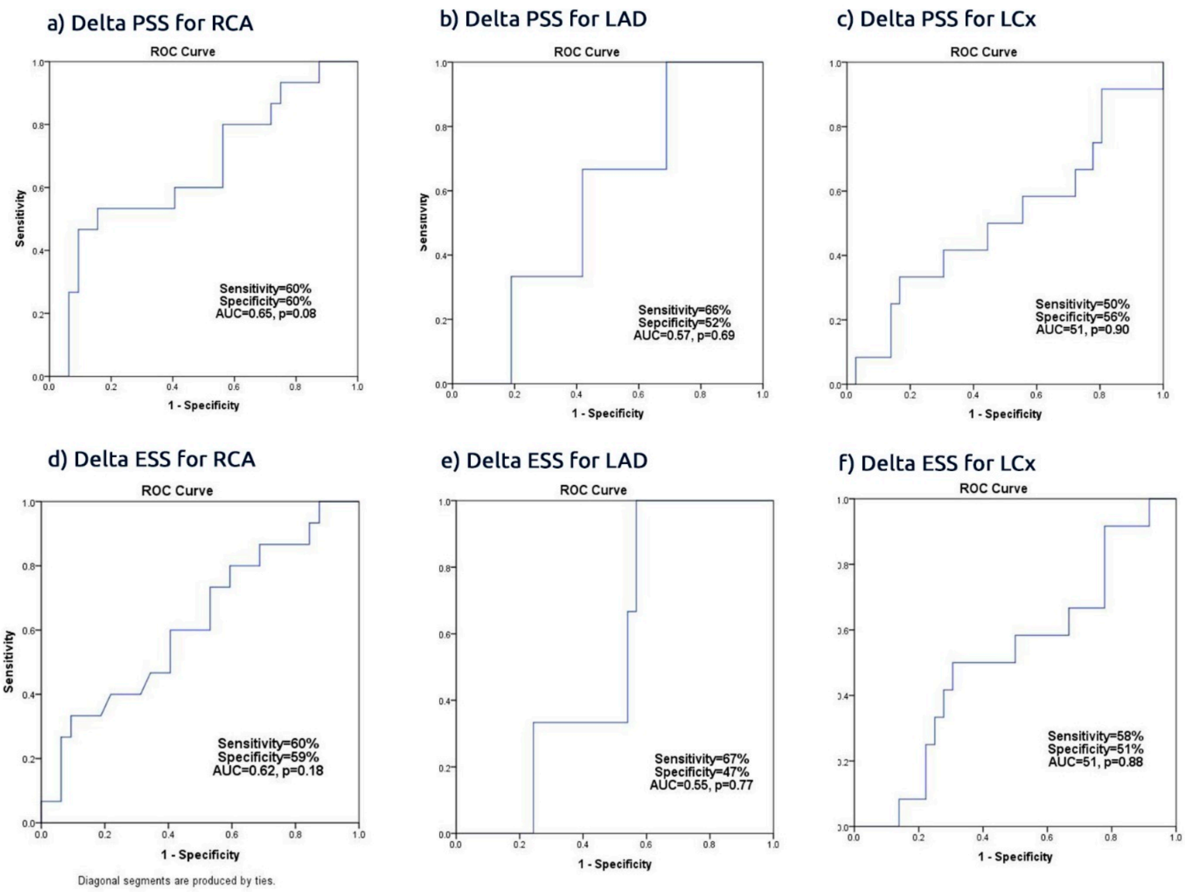

Supplement 3. Delta PSS and ESS in predicting territories supplied for: a) Delta PSS for RCA; b) Delta PSS for LAD c) Delta PSS for LCx; d) ESS for RCA; e) Delta ESS for LAD; f) Delta ESS for LCx.

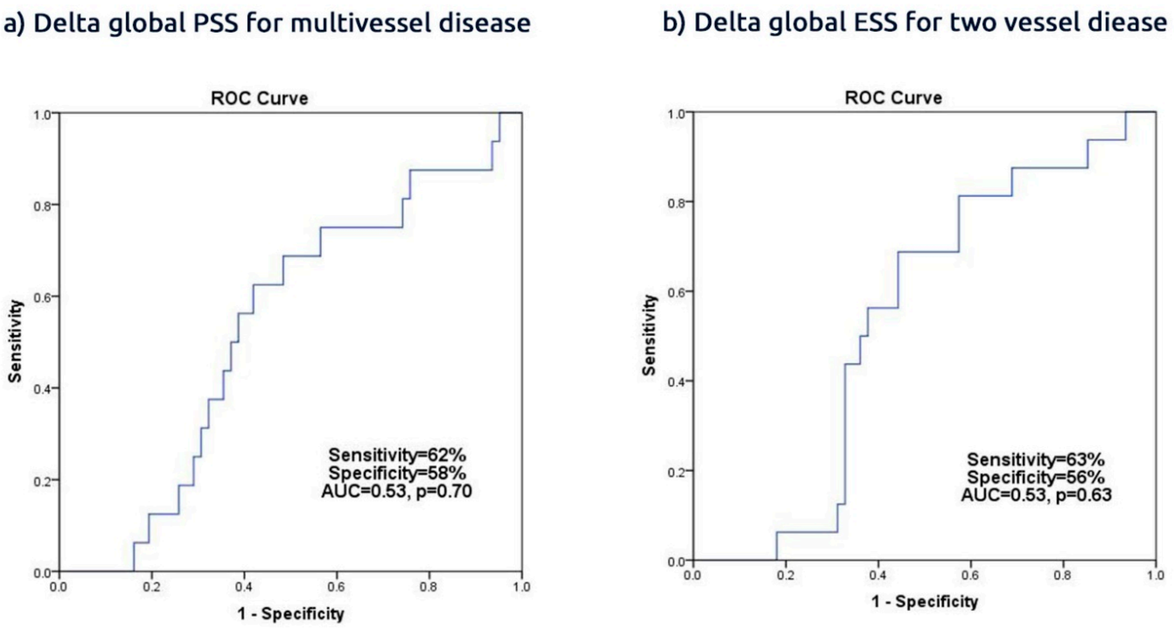

Supplement 4. Delta PSS and ESS in predicting multi vessel disease: a) Delta PSS; b) Delta ESS.

Supplement 5. Delta regional of SR supplied for territories arteries; basal vs. apical.

Variable	R	P
**WMA for LAD disease**		
Basal	0.26	0.02
Apical	0.22	0.04
**WMA for LCx disease**		
Basal	0.40	0.002
Apical	0.39	0.003
**WMA for RCA disease**		
Basal	0.43	0.001
Apical	0.37	0.01

WMA basal for LAD: Peak systolic SR basal-anterior; WMA apical for LAD: Peak systolic SR apico-septal, Peak systolic SR apico-anterior; WMA basal for LCx: Peak systolic SR basal-septal, Peak systolic SR basal-lateral; WMA apical for LCx: Peak systolic SR apico-lateral; WMA basal for RCA: Peak systolic SR basal-inferior, Peak systolic SR basal-posterior; WMA apical for RCA: Peak systolic apico-inferior.
